# Suppression of Obesity by an Intestinal Helminth through Interactions with Intestinal Microbiota

**DOI:** 10.1128/IAI.00042-19

**Published:** 2019-05-21

**Authors:** Chikako Shimokawa, Seiji Obi, Mioko Shibata, Alex Olia, Takashi Imai, Kazutomo Suzue, Hajime Hisaeda

**Affiliations:** a Department of Parasitology, Graduate School of Medicine, Gunma University, Gunma, Japan; b Department of Parasitology, National Institute of Infectious Diseases, Tokyo, Japan; University of Pennsylvania

**Keywords:** helminth, immunity, obesity, microbiota

## Abstract

Obesity is increasingly causing lifestyle diseases in developed countries where helminthic infections are rarely seen. Here, we investigated whether an intestinal nematode, Heligmosomoides polygyrus, has a suppressive role in diet-induced obesity in mice. Infection with H. polygyrus suppressed weight gain in obese mice, which was associated with increased uncoupling protein 1 (UCP1) expression in adipocytes and a higher serum norepinephrine (NE) concentration. Blocking interactions of NE with its receptor on adipocytes resulted in the failure to prevent weight gain and to enhance UCP1 expression in obese mice infected with H. polygyrus, indicating that NE is responsible for the protective effects of H. polygyrus on obesity. In addition to sympathetic nerve-derived NE, the intestinal microbiota was involved in the increase in NE. Infection with H. polygyrus altered the composition of intestinal bacteria, and antibiotic treatment to reduce intestinal bacteria reversed the higher NE concentration, UCP1 expression, and prevention of the weight gain observed after H. polygyrus infection. Our data indicate that H. polygyrus exerts suppressive roles on obesity through modulation of microbiota that produce NE.

## Abstract (precis)

Obesity is increasingly causing lifestyle diseases in developed countries where helminthic infections are rarely seen. Here, we investigated whether an intestinal nematode, Heligmosomoides polygyrus, has a suppressive role in diet-induced obesity in mice.

## INTRODUCTION

Obesity causes lifestyle diseases such as hypertension, diabetes, and dyslipidemia (1). Excess energy consumption is an important cause of obesity. The overall body energy balance is maintained by adjusting excess or deficient energy in conjunction with energy intake and output. One such metabolic control is present in adipose tissues. Adipose tissue is classified as white or brown (2), with each having different characteristics. White adipose tissue accumulates excessive lipids as triglycerides, while brown adipose tissue consumes fatty acids as heat (3). Brown adipose tissues contain two distinct types of thermogenic adipocytes: classical brown adipocytes and beige or bright adipocytes. A major goal of research on obesity is to understand the factors that activate thermogenic adipocytes, which activate energy consumption, thus preventing obesity.

Uncoupling protein 1 (UCP1) is an integral membrane protein expressed in the mitochondria of brown (4) and beige (5) adipocytes, and it uncouples oxidative phosphorylation. When UCP1 is activated, energy generated by the lipolysis of fatty acids and glucose is directly converted to heat, without being directed to ATP synthesis, and the heat then dissipates (6). Thus, this molecule is crucial for energy expenditure. Heat production by UCP1 is controlled by norepinephrine (NE), which is released from sympathetic nerves that are densely distributed in brown fat. Fatty acids are generated through lipolysis that is induced by NE acting on its receptor and driving activation of adipocyte lipases, including adipose triglyceride lipase and hormone-sensitive lipase. The fatty acid produced is oxidatively decomposed and activates UCP1. When NE acts on white adipocytes, fat decomposition similarly occurs, but the fatty acid produced is released into the blood and is consumed by brown adipocytes and muscle. A characteristic of beige adipocytes is the dynamic regulation of UCP1 by external stimuli. β3-Adrenergic receptor (β3AdR) agonists induced the marked expression of UCP1 in beige adipocytes (5).

Recent studies demonstrated a close relationship between intestinal microbiota composition and several diseases, including metabolic, gastrointestinal, and inflammatory diseases (7, 8). Additionally, obesity is associated with low gut microbiota diversity, and it may alter the composition of certain bacteria in humans and animal models (9). Many factors that affect the onset of obesity are associated with modulation of the microbiota (10, 11). The intestinal microbiota is associated with psychiatric diseases including depression and autism, and previous studies reported that intestinal bacteria communicated with the central nervous system to stimulate the production of neurotransmitters and hormones, including serotonin, dopamine, and γ-amino butyric acid (12–14).

The incidence of obesity has increased, especially in developed countries where helminthic infections have almost been eliminated (15). Several lines of evidence indicate an inverse correlation between helminthic infections and obesity as well as inflammation-mediated disorders (16, 17), suggesting that helminths may have suppressive effects on these diseases. However, protective mechanisms involved in how helminths suppress obesity are largely unknown. Given that intestinal helminths modulate gut microbiota, the current study investigated the effects of an intestinal nematode on obesity in mice fed a high-fat diet (HFD) by focusing on the gut microbiota. We found that helminthic infection affected gut bacteria, resulting in increased NE production that upregulated UCP1 in adipose tissues.

## RESULTS

### Infection with Heligmosomoides polygyrus reduces established obesity.

**FIG 1:**
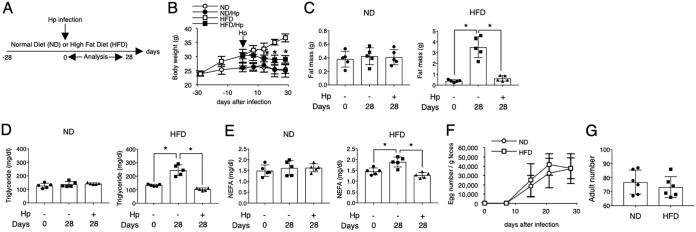
Reduction of preexisting obesity in mice infected with Heligmosomoides polygyrus (Hp). (A) Experimental protocol for generating obese mice. Mice fed a high-fat diet (HFD) for 28 days were orally infected with 200 L3 H. polygyrus larvae, and the HFD was continued for the entire experimental period. A normal diet (ND) was used as a control. All analyses were performed 28 days after infection unless otherwise mentioned. (B) Body weight of mice fed an HFD or an ND in the presence or absence of H. polygyrus infection was periodically monitored. The weight of epididymal adipose tissue (C) and the blood concentration of triglycerides (D) and nonesterified fatty acid (NEFA) (E) in the indicated groups were measured. (F and G) The number of H. polygyrus eggs per gram of feces was monitored weekly, and the number of adult worms in the small intestine was counted 28 days after infection in mice fed an HFD or an ND. All values are presented as the means ± standard deviations of five or six mice in one representative experiment. Symbols in scatter graphs with bars represent data from an individual animal. Similar results were obtained from three replicate experiments. *, *P* < 0.05, by Student's *t* test or two-way ANOVA.

To investigate whether infection with Heligmosomoides polygyrus has therapeutic effects on established obesity, we used mice fed an HFD for 4 weeks as infection hosts (Fig. 1A). This diet resulted in an increase in body weight up to 20% and an increase in fat mass and dyslipidemia (Fig. 1B to E), and these mice were considered obese. Before analysis, we confirmed that feeding with an HFD had no adverse effect on H. polygyrus infection. Obese mice harbored adult worms and produced eggs at levels comparable to those of mice fed a normal diet (ND) (Fig. 1F and G). Continuously feeding obese mice an HFD accelerated the increase in body weight. Infection of obese mice with H. polygyrus decreased body weight and improved dyslipidemia (Fig. 1B to E). These results suggested that H. polygyrus infection has preventive and therapeutic effects on existing obesity.

### H. polygyrus-mediated reduction in obesity is associated with UCP1 expression induced via NE.

**FIG 2:**
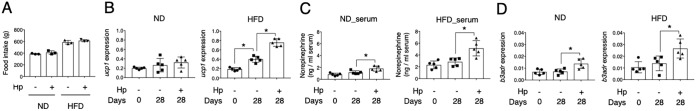
Potential causes of obesity correction during H. polygyrus infection. (A) The amount of food intake by each group was measured. Consumption of the diet by each group comprised of five mice housed in one cage was estimated by subtracting the weight of food remaining after 1 week of feeding from the original weight of the food provided, and this measurement was repeated for 4 weeks. Values represent the means ± standard deviations of three individual experiments. (B) mRNA encoding UCP1 in adipocytes obtained from epididymal adipose tissues of the indicated groups. The expression level relative to GAPDH mRNA is presented. (C) The NE concentration in the blood obtained from the indicated group was determined by ELISA. (D) mRNA encoding β3AdR was quantified as described for panel B. All values in panels B, C, and D are presented as the means ± standard deviations of five mice in one representative experiment. Symbols in scatter graphs with bars represent data from an individual animal. Similar results were obtained from three replicate experiments. *, *P* < 0.05, using a two-way ANOVA.

**FIG 3:**
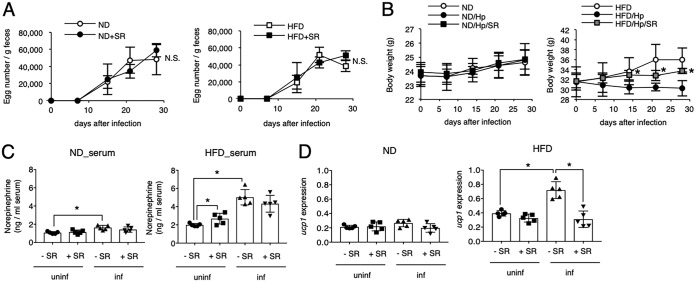
Critical roles of NE in the anti-adiposity effects of H. polygyrus infection. (A) The number of H. polygyrus eggs per gram of feces in mice treated with a β3AdR antagonist, SR59230A (SR), was monitored weekly. (B) Body weight of obese mice infected with H. polygyrus in the presence of SR was monitored. (C and D) The serum NE concentration and UCP1 mRNA expression level in the indicated mouse group were evaluated as described in the legend of Fig. 2. All values are presented as the means ± standard deviations of five mice in one representative experiment. Symbols in scatter graphs with bars represent data from an individual animal. *, *P* < 0.05; N.S., not significant (using a two-way ANOVA). uninf, uninfected; inf, infected.

Because food intake in obese mice was not influenced by infection with H. polygyrus (Fig. 2A), weight loss might be a result of excess calorie consumption rather than of a reduction of calorie intake. We postulated that, in addition to a marked decrease in fat mass (Fig. 1C), H. polygyrus infection might promote energy generation in adipose tissues, which is controlled by UCP1 expression in adipocyte mitochondria. Thus, UCP1 mRNA expression in adipocytes obtained from H. polygyrus-infected obese mice was quantified (Fig. 2B). Although UCP1 was substantially upregulated even in the absence of H. polygyrus as obesity proceeded, infection with H. polygyrus induced significantly more UCP1 expression. UCP1 expression in adipocytes is regulated by sympathetic innervation through the production of NE (18). In parallel with UCP1 expression, obese mice infected with H. polygyrus showed the highest concentration of NE in the blood (Fig. 2C). Released NE acts on β3AdR expressed on white adipocytes. We also analyzed the mRNA expression of β3AdR in adipocytes, and the same trends as for UCP1 and NE were observed: obesity increased β3AdR expression, and infection with H. polygyrus had additional effects (Fig. 2D). Infection of nonobese ND-fed mice with H. polygyrus resulted in a slight increase in NE levels and the expression of β3AdR, but it had no effect on UCP1 expression (Fig. 2B to D). These results suggest that UCP1 expression is dependent on NE-β3AdR interactions, which may be responsible for the reduction of obesity in obese mice infected with H. polygyrus. To test this possibility, a selective antagonist of β3AdR, SR59230 (19), was administered to H. polygyrus-infected obese mice. Blockade of β3AdR did not alter the ability to produce eggs in either obese or nonobese mice (Fig. 3A). Mice treated with SR59230 failed to suppress weight gain even in the presence of H. polygyrus (Fig. 3B). Although SR59230 did not affect the NE concentration, it markedly suppressed the increased UCP1 mRNA expression (Fig. 3C and D). In mice fed an ND, there was no change in body weight, NE concentration, or UCP1 expression (Fig. 3B to D). Thus, the protective role of H. polygyrus infection against obesity is dependent on NE, presumably by inducing UCP1 for energy expenditure in adipocytes.

### Neurogenic NE contributes to the H. polygyrus-mediated reduction of obesity.

**FIG 4:**
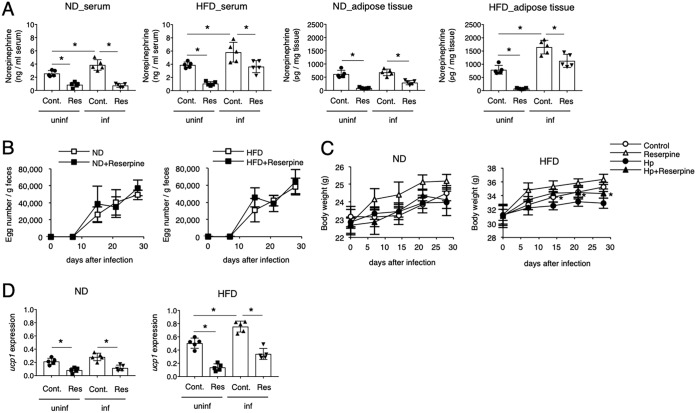
Involvement of neurogenic NE in the H. polygyrus-mediated suppression of weight gain. Serum and adipose tissue NE concentrations (A), the number of H. polygyrus eggs per gram of feces (B), body weight (C), and UCP1 mRNA expression (D) in mice treated with reserpine (Res) were monitored. All values are presented as the means ± standard deviations of five mice in one representative experiment. Symbols in scatter graphs with bars represent data from an individual animal. Cont, control. *, *P* < 0.05, using a two-way ANOVA.

We next analyzed the mechanisms involved in the production of NE during H. polygyrus infection. Infection of obese mice with H. polygyrus resulted in an increase in NE concentrations in the serum and adipose tissues (Fig. 4A). Because sympathetic innervation is the major source of NE in adipose tissues (20), H. polygyrus-infected obese mice were subjected to chemical denervation by treatment with reserpine. This agent blocks the intake of catecholamine into presynaptic vesicles and therefore allows assessment of nervous system involvement in the suppression of obesity. Mice treated with reserpine produced similar numbers of eggs as untreated mice (Fig. 4B). In the absence of infection, treatment with reserpine significantly reduced the NE concentration and expression of UCP1 in adipose tissue, resulting in increased body weight in mice fed with an ND and an HFD (Fig. 4A, C, and D). Similarly, H. polygyrus-infected mice treated with reserpine exhibited lower NE concentrations and lower expression levels of UCP1 (Fig. 4A and C). Suppression of obesity observed in H. polygyrus-infected obese mice was attenuated in the presence of reserpine (Fig. 4D), indicating the involvement of neurogenic NE in the expression of UCP1 and the suppression of weight gain.

### The intestinal microbiota is involved in the H. polygyrus-mediated reduction of obesity.

**FIG 5:**
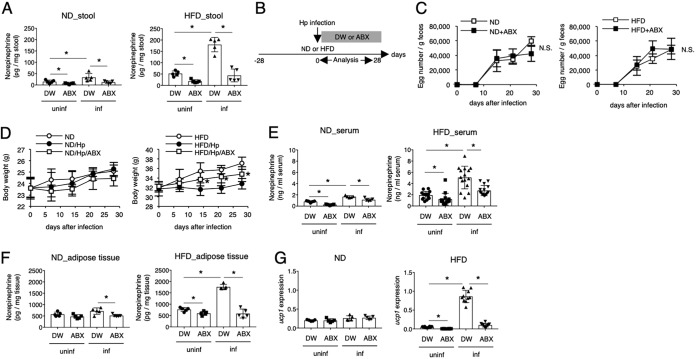
Involvement of intestinal microbiota in NE production in obese mice infected with H. polygyrus. (A) Fecal NE concentrations in the indicated mice were analyzed. (B) Scheme for the treatment of H. polygyrus-infected obese mice with antibiotics (ABX). The number of H. polygyrus eggs per gram of feces (C), body weight (D), NE concentration in serum (E) and adipose tissue (F), and UCP1 mRNA expression (G) in the indicated mouse groups were evaluated as described in the legends of Fig. 2 and 4. All values are presented as the means ± standard deviations of more than five mice in one representative experiment. Symbols in scatter graphs with bars represent data from an individual animal. *, *P* < 0.05, using a two-way ANOVA. DW, distilled water.

Although treatment of uninfected mice with reserpine completely suppressed NE production in adipose tissues and serum, obese mice infected with H. polygyrus still contained substantial amounts of NE after treatment with reserpine (Fig. 4A), suggesting the existence of other sources of NE besides sympathetic nerves. Thus, we next addressed how H. polygyrus induces NE production by focusing on the intestinal microbiota (i) because H. polygyrus parasitizes small intestines and affects the microbiota (16, 17) and (ii) because several neurotransmitters, including NE, originate from the microbiota (21, 22). In addition, infection of obese mice with H. polygyrus increased NE levels in the feces (Fig. 5A). To this end, obese mice infected with H. polygyrus were orally treated with antibiotics to reduce the intestinal microbiota (Fig. 5B). This treatment did not affect the parasitism of H. polygyrus as assessed by egg production (Fig. 5C). Treatment with antibiotics decreased both the NE concentration and expression of UCP1 in H. polygyrus-infected obese mice, resulting in the attenuated suppression of weight gain (Fig. 5D to G). Thus, the microbiota has a crucial role in the H. polygyrus-mediated prevention of obesity.

### Infection with H. polygyrus changes the intestinal microbiota in obese mice.

**FIG 6:**
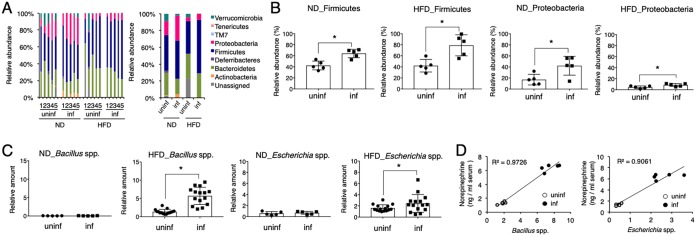
Changes in intestinal microbiota in obese mice infected with H. polygyrus. (A) Composition of intestinal microbiota at the phylum level in the indicated mouse groups is shown. Each bar in the left and right panels depicts the composition of an individual mouse and the mean value from five mice, respectively. (B) The frequency of *Firmicutes* and *Proteobacteria* in mice represented by the data in panel A is shown. (C) The abundances of *Bacillus* and *Escherichia* species in the overall intestinal microbiota in mice 28 days after infection were calculated based on quantitative PCR, as described below. Relative abundance (%) = 2^(^*^CT^* ^universal 16S −^ *^CT^* ^specific 16S)^ × 100, where *C_T_* is threshold cycle. All values are presented as the means ± standard deviations of more than five mice in one representative experiment. *, *P* < 0.05, using a two-way ANOVA. (D) The representative coplotted NE concentration and abundance of genera in five individual mice in the presence or absence of H. polygyrus infection 28 days after infection are presented. *R*^2^, the correlation coefficient.

To investigate changes in the microbiota responsible for the increase in NE production during infection with H. polygyrus, the fecal microbiota of H. polygyrus-infected mice was comprehensively analyzed. Feeding with the HFD alone altered the composition of microbiota, characterized by a decrease in *Proteobacteria* and an increase in unassigned bacteria (Fig. 6A). Mice infected with H. polygyrus contained more representatives of the *Firmicutes* and *Proteobacteria* than uninfected mice independent of diet they were fed (Fig. 6A and B). Previous studies reported that two bacterial genera, *Bacillus* and *Escherichia* belonging to *Firmicutes* and *Proteobacteria*, respectively, produced NE in the intestines (23). Quantitative real-time PCR analyses of microbiota revealed that infection of obese mice with H. polygyrus significantly increased *Bacillus* and *Escherichia* species (Fig. 6C). In addition, the NE concentration in H. polygyrus-infected obese mice was closely correlated with the amounts of those bacteria (Fig. 6D). These results suggest that infection with H. polygyrus modulates intestinal bacteria to produce NE, which is responsible for limiting weight gain.

## DISCUSSION

In this study, we demonstrated that H. polygyrus suppressed HFD-induced obesity. Mechanistically, H. polygyrus affected the composition of the intestinal microbiota to increase NE, resulting in enhanced UCP1 expression in adipose tissues. Because stimulation with NE was reported to induce beige adipocytes from adipocyte progenitors associated with UCP1 expression (5), these thermogenic adipocytes might be induced during H. polygyrus infection.

Recently, Su et al. reported the preventive effects of H. polygyrus infection on HFD-induced obesity, focusing on distinct immune responses (24). Alternative activated macrophages (AAMs) induced during H. polygyrus infection suppressed insulin resistance and inflammation associated with obesity and enhanced UCP1 expression in adipose tissues. We also think that the decisive effector that prevents obesity is increased UCP1 expression. However, we revealed different mechanisms from those of Su et al. involving AAMs that upregulated UCP1 expression. This difference may be due to difference in experimental system, and our system elicited mild obesity without causing hyperglycemia or insulin resistance. Although we did not investigate immune responses, mild obesity was not associated with inflammation, indicating that it may be regulated in ways other than by immune responses.

Our results using a β3AdR antagonist demonstrated that NE plays a crucial role in UCP1 expression. Chemical denervation using reserpine increased body weight even in the absence of H. polygyrus infection, indicating that neurogenic NE primarily regulates the induction of UCP1 under physiological conditions. In addition to neurogenic NE, intestinal microbiota contributed to increased NE levels in obese mice infected with H. polygyrus. Because H. polygyrus resides in the small intestine, it is thought to affect the intestinal microbiota. Several studies demonstrated that the intestinal flora composition was altered after H. polygyrus infection (25, 26). Here, we observed that H. polygyrus infection altered the gut microbiota composition, and, specifically, more *Bacillus* and *Escherichia* species were detected, both of which are known to generate NE (14, 27). Other bacterial products such as short-chain fatty acids are known to have anti-obesity effects, and some bacteria are associated with obesity (28); therefore, additional effects caused by changes in microbiota may contribute to the reduction of obesity. Thus, comprehensive analyses of the intestinal microbiota are required to understand metabolic homeostasis better during H. polygyrus infection.

The question arises as to whether the induction of NE is favorable to H. polygyrus parasitism. The expulsion of intestinal worms depends on the production of mucin and peristaltic movements controlled by parasympathetic nerves. Thus, the activation of sympathetic nerves may help intestinal parasites to settle by suppressing intestinal movement. However, this seems unlikely because egg production was not decreased by treatment with the β3AdR agonist or antibiotics (Fig. 3A and 5C). We did not address how H. polygyrus modulates the microbiota at the molecular level. Further analyses to determine which molecules are involved will be valuable to treat or prevent obesity, such as by using prebiotics and probiotics.

## MATERIALS AND METHODS

### Mice.

Male C57BL/6J mice were purchased from Japan SLC (Hamamatsu, Japan), maintained under specific pathogen-free conditions, and used for experiments at 10 to 12 weeks of age. For experimental feeding, an HFD containing 60% fat (HFD-60; Oriental Yeast Corporation, Tokyo, Japan) and a control normal diet (AIN-93M; Oriental Yeast Corporation) were used. All animal experiments were reviewed and approved by the Committee for Ethics on Animal Experiments at the Graduate School of Gunma University (approval number 16-041) and were conducted under the control of the Guidelines for Animal Experiments in the Graduate School of Gunma University and in accordance with Law No. 105 and Notification No. 6 of the Japanese Government.

### H. polygyrus infection.

Infectious stage III H. polygyrus larvae (L3) were prepared as previously described (29) and stored at 4°C until use. Mice were inoculated orally with 200 L3 larvae using gastric intubation. Eggs in feces were detected using a microscope to confirm successful infection.

### Sample collection.

Blood was taken from the mice via cardiac puncture under anesthesia, and mice were sacrificed by cervical dislocation. Epididymal adipose tissue was aseptically removed, and adipocytes were purified as previously reported (30). The adipose tissue weight was then measured. Serum samples were separated from the collected blood for analyses. In some experiments, all adult worms recovered from the small intestine of mice infected with H. polygyrus were counted.

### Serum analysis.

Serum samples were analyzed for triglyceride and nonesterified fatty acid (NEFA) using LabAssay (Wako, Tokyo, Japan) and for NE using a standard enzyme-linked immunosorbent assay (ELISA), in accordance with the manufacturer’s instructions (ISM, Tokyo, Japan).

### Real-time RT-PCR analysis.

Total RNA was extracted from purified adipocytes using an RNeasy Mini kit (Qiagen, Hilden, Germany) and reverse transcribed using ReverTra Ace (Toyobo, Osaka, Japan) to synthesize cDNA. Resultant cDNA expressing the genes of interest was quantified, by real-time reverse transcription-PCR (RT-PCR) using SYBR green (TaKaRa Bio, Shiga, Japan), relative to the level of mRNA encoding glyceraldehyde-3-phosphate dehydrogenase (GAPDH) in accordance with the manufacturer’s protocol. The specific primer pairs were as follows: for *Ucp1*, 5′-ACTGCCACACCTCCAGTCATT-3′ and 5′-CTTTGCCTCACTCAGGATTGG-3′; for *Adrb3*, 5′-TCGACATGTTCCTCCACCAA-3′ and 5′-GATGGTCCAAGATGGTGCTT-3′; and for *Gapdh*, 5′-TGTGTCCGTCGTGGATCTGA-3′ and 5′-TTGCTGTTGAAGTCGCAGGAG-3′.

### Receptor blockade.

For β3 adrenergic receptor blockade, 5 mg/kg body weight of SR59230 (Sigma, St. Louis, MO, USA) was intraperitoneally injected every other day for 28 days (31).

### Reserpine injection.

Reserpine (0.5 mg/kg body weight) was intraperitoneally injected 1 day before and 3 and 5 days after H. polygyrus infection.

### Antibiotic treatment.

To reduce gut bacteria, obese mice infected with H. polygyrus were administered a mixture of ampicillin (1 g/liter), vancomycin (0.5 g/liter), neomycin (1 g/liter), and metronidazole (1 g/liter) in their drinking water for 28 days.

### Gut microbiota analysis by 16S rRNA sequencing.

Fecal samples collected from mice were immediately frozen in liquid nitrogen and stored at −80°C. Fecal DNA extraction was performed according a previous study with minor modifications (32). A grain of mouse feces was suspended with sterilized sticks in 475 μl of TE10 buffer containing 10 mM Tris-HCl (pH 8.0) and 10 mM EDTA. The fecal suspension was incubated with 15 mg/ml lysozyme (Wako) at 37°C for 1 h. A final concentration of 2,000 units/ml of purified achromopeptidase (Wako) was added and then incubated at 37°C for 30 min. We added 1% (wt/vol) sodium dodecyl sulfate and 1 mg/ml proteinase K (Merck Japan, Tokyo, Japan) to the suspension and incubated it at 55°C for 1 h. After centrifugation of the suspension, bacterial DNA was purified using phenol-chloroform-isoamyl alcohol (25:24:1) solution. DNA was precipitated by adding ethanol and sodium acetate. RNase A (Wako) was added to bacterial DNA in TE buffer to a final concentration 1 mg/ml. To remove fragmented low-molecular-weight DNA, precipitation with polyethylene glycol (PEG) 6000 was performed after RNase treatment.

The V4 variable region (residues 515F to 806R) was sequenced on an Illumina MiSeq, according to the method of Kozich et al. (33). Each reaction mixture contained 15 pmol of each primer, 0.2 mM deoxyribonucleoside triphosphates, 5 μl of 10× Ex Taq HS buffer, 1.25 U Ex Taq HS polymerase (TaKaRa Bio), 50 ng of extracted DNA, and sterilized water to a final volume of 50 μl. PCR conditions were as follows: 95°C for 2 min, 25 cycles of 95°C for 20 s, 55°C for 15 s, and 72°C for 5 min, followed by 72°C for 10 min. The PCR product was purified by AMPure XP (Beckman Coulter, Brea, CA, USA) and quantified using a Quant-iT PicoGreen double-stranded DNA (dsDNA) assay kit (Life Technologies Japan, Tokyo, Japan). Mixed samples were prepared by pooling approximately equal amounts of PCR amplicons from each sample. The pooled library was analyzed with an Agilent High Sensitivity DNA kit on an Agilent 2100 Bioanalyzer (Agilent Technologies, CA, USA). Real-time PCR for quantification was performed on a pooled library using a KAPA Library Quantification kit for Illumina according to the manufacturer’s protocols. Based on the quantification, the sample library was denatured and diluted. A sample library with 20% denatured PhiX spike-in was sequenced by MiSeq using a 500-cycle kit. We obtained 2- by 250-bp paired-end reads. Taxonomic assignments and the estimation of relative abundances from sequencing data were performed using the analysis pipeline of the QIIME software package (34).

### Quantification of fecal microbiota with real-time PCR.

DNA from mouse stools was extracted using a Stool Mini kit (Qiagen). Genes encoding 16S rRNA were quantified using a reverse transcription-quantitative PCR (RT-qPCR) kit (Qiagen). The specific primer pairs were as follows: for *Escherichia* spp., 5′-GTTAATACCTTTGCTCATTGA-3′ and 5′-ACCAGGGTATCTAATCCTGTT-3′ (35); for *Bacillus* spp., 5′-CAGTAGGGAATCTTCCGCAATG-3′ and 5′-AGCCGTGGCTTTCTGGT-3′ (36). A universal primer pair for all bacteria was also used: 5′-GTGGTGCACGGCTGTCGTCA-3′ and 5′-ACGTCATCCACACCTTCCTC-3′ (37).

### Statistical analysis.

Group means were compared by two-way analysis of variance (ANOVA), followed by Tukey’s *post hoc* test or two-tailed Student's *t* test. Probability values below 0.05 were considered statistically significant.

## References

[1] Halpern A, Mancini MC, Magalhaes ME, Fisberg M, Radominski R, Bertolami MC, Bertolami A, de Melo ME, Zanella MT, Queiroz MS, Nery M 2010 Metabolic syndrome, dyslipidemia, hypertension and type 2 diabetes in youth: from diagnosis to treatment. Diabetol Metab Syndr 2:55. doi:10.1186/1758-5996-2-55. 20718958 PMC2939537

[2] Kajimura S 2017 Adipose tissue in 2016: Advances in the understanding of adipose tissue biology. Nat Rev Endocrinol 13:69–70. doi:10.1038/nrendo.2016.211. 28051117 PMC5465959

[3] Sanchez-Gurmaches J, Hung C-M, Guertin DA 2016 Emerging complexities in adipocyte origins and identity. Trends Cell Biol 26:313–326. doi:10.1016/j.tcb.2016.01.004. 26874575 PMC4844825

[4] Walker JE, Runswick MJ 1993 The mitochondrial transport protein superfamily. J Bioenerg Biomembr 25:435–446. doi:10.1007/BF01108401. 8132484

[5] Kajimura S, Spiegelman BM, Seale P 2015 Brown and beige fat/physiological roles beyond heat generation. Cell Metab 22:546–559. doi:10.1016/j.cmet.2015.09.007. 26445512 PMC4613812

[6] Busiello RA, Savarese S, Lombardi A 2015 Mitochondrial uncoupling proteins and energy metabolism. Front Physiol 6:36. doi:10.3389/fphys.2015.00036. 25713540 PMC4322621

[7] Backhed F, Ley RE, Sonnenburg JL, Peterson DA, Gordon JI 2005 Host-bacterial mutualism in the human intestine. Science 307:1915–1920. doi:10.1126/science.1104816. 15790844

[8] Neish AS 2009 Microbes in gastrointestinal health and disease. Gastroenterology 136:65–80. doi:10.1053/j.gastro.2008.10.080. 19026645 PMC2892787

[9] Wolf KJ, Lorenz RG 2012 Gut microbiota and obesity. Curr Obes Rep 1:1–8. doi:10.1007/s13679-011-0001-8. 23106036 PMC3478901

[10] Baothman OA, Zamzami MA, Taher I, Abubaker J, Abu-Farha M 2016 The role of gut microbiota in the development of obesity and diabetes. Lipids Health Dis 15:108. doi:10.1186/s12944-016-0278-4. 27317359 PMC4912704

[11] Diamant M, Blaak EE, de Vos WM 2011 Do nutrient-gut-microbiota interactions play a role in human obesity, insulin resistance and type 2 diabetes? Obes Rev 12:272–281. doi:10.1111/j.1467-789X.2010.00797.x. 20804522

[12] Strandwitz P 2018 Neurotransmitter modulation by the gut microbiota. Brain Res 1693:128–133. doi:10.1016/j.brainres.2018.03.015. 29903615 PMC6005194

[13] Sarkar A, Lehto SM, Harty S, Dinan TG, Cryan JF, Burnet PWJ 2016 Psychobiotics and the manipulation of bacteria-gut-brain signals. Trends Neurosci 39:763–781. doi:10.1016/j.tins.2016.09.002. 27793434 PMC5102282

[14] Dinan TG, Stilling RM, Stanton C, Cryan JF 2015 Collective unconscious: how gut microbes shape human behavior. J Psychiatr Res 63:1–9. doi:10.1016/j.jpsychires.2015.02.021. 25772005

[15] Bhurosy T, Jeewon R 2014 Overweight and obesity epidemic in developing countries: a problem with diet, physical activity, or socioeconomic status? ScientificWorldJournal 2014:964236. doi:10.1155/2014/964236. 25379554 PMC4212551

[16] Weinstock JV, Elliott DE 2014 Helminth infections decrease host susceptibility to immune-mediated diseases. J Immunol 193:3239–3247. doi:10.4049/jimmunol.1400927. 25240019 PMC4244645

[17] Wang LJ, Cao Y, Shi HN 2008 Helminth infections and intestinal inflammation. World J Gastroenterol 14:5125–5132. doi:10.3748/wjg.14.5125. 18777588 PMC2744001

[18] Inokuma K, Ogura-Okamatsu Y, Toda C, Kimura K, Yamashita H, Saito M 2005 Uncoupling protein 1 is necessary for norepinephrine-induced glucose utilization in brown adipose tissue. Diabetes 54:1385–1391. doi:10.2337/diabetes.54.5.1385. 15855324

[19] Galitzky J, Langin D, Verwaerde P, Montastruc JL, Lafontan M, Berlan M 1997 Lipolytic effects of conventional beta 3-adrenoceptor agonists and of CGP 12,177 in rat and human fat cells: preliminary pharmacological evidence for a putative beta 4-adrenoceptor. Br J Pharmacol 122:1244–1250. doi:10.1038/sj.bjp.0701523. 9401793 PMC1565062

[20] Bartness Lab J, Langin D, Verwaerde P, Montastruc JL, Lafontan M, Berlan M 2014 Neural innervation of white adipose tissue and the control of lipolysis. Front Neuroendocrinol 35:473–493. doi:10.1016/j.yfrne.2014.04.001. 24736043 PMC4175185

[21] Holzer P, Farzi A 2014 Neuropeptides and the microbiota-gut-brain axis. Adv Exp Med Biol 817:195–219. doi:10.1007/978-1-4939-0897-4_9. 24997035 PMC4359909

[22] Galland L 2014 The gut microbiome and the brain. J Med Food 17:1261–1272. doi:10.1089/jmf.2014.7000. 25402818 PMC4259177

[23] Mazzoli R, Pessione E 2016 The neuro-endocrinological role of microbial glutamate and GABA signaling. Front Microbiol 7:1934. doi:10.3389/fmicb.2016.01934. 27965654 PMC5127831

[24] Su CW, Chen CY, Li Y, Long SR, Massey W, Kumar DV, Walker WA, Shi HN 2018 Helminth infection protects against high fat diet-induced obesity via induction of alternatively activated macrophages. Sci Rep 8:4607. doi:10.1038/s41598-018-22920-7. 29545532 PMC5854586

[25] Zaiss MM, Rapin A, Lebon L, Dubey LK, Mosconi I, Sarter K, Piersigilli A, Menin L, Walker AW, Rougemont J, Paerewijck O, Geldhof P, McCoy KD, Macpherson AJ, Croese J, Giacomin PR, Loukas A, Junt T, Marsland BJ, Harris NL 2015 The intestinal microbiota contributes to the ability of helminths to modulate allergic inflammation. Immunity 43:998–1010. doi:10.1016/j.immuni.2015.09.012. 26522986 PMC4658337

[26] Walk ST, Blum AM, Ewing SA, Weinstock JV, Young VB 2010 Alteration of the murine gut microbiota during infection with the parasitic helminth Heligmosomoides polygyrus. Inflamm Bowel Dis 16:1841–1849. doi:10.1002/ibd.21299. 20848461 PMC2959136

[27] Cryan JF, Dinan TG 2012 Mind-altering microorganisms: the impact of the gut microbiota on brain and behaviour. Nat Rev Neurosci 13:701–712. doi:10.1038/nrn3346. 22968153

[28] Chandra KC 2015 New-found link between microbiota and obesity. World J Gastrointest Pathophysiol 6:110–119. doi:10.4291/wjgp.v6.i4.110. 26600968 PMC4644874

[29] Shimokawa C, Kanaya T, Hachisuka M, Ishiwata K, Hisaeda H, Kurashima Y, Kiyono H, Yoshimoto T, Kaisho T, Ohno H 2017 Mast cells are crucial for induction of group 2 innate lymphoid cells and clearance of helminth infections. Immunity 46:863–874. doi:10.1016/j.immuni.2017.04.017. 28514691

[30] Rodbell M 1964 Localization of lipoprotein lipase in fat cells of rat adipose tissue. J Biol Chem 239:753–755. 14154450

[31] Vasina V, Giannone F, Domenicali M, Latorre R, Berzigotti A, Caraceni P, Zoli M, De Ponti F, Bernardi M 2012 Portal hypertension and liver cirrhosis in rats: effect of the β3-adrenoceptor agonist SR58611A. Br J Pharmacol 167:1137–1147. doi:10.1111/j.1476-5381.2012.02074.x. 22708587 PMC3492993

[32] Atarashi K, Tanoue T, Ando M, Kamada N, Nagano Y, Narushima S, Suda W, Imaoka A, Setoyama H, Nagamori T, Ishikawa E, Shima T, Hara T, Kado S, Jinnohara T, Ohno H, Kondo T, Toyooka K, Watanabe E, Yokoyama S-i, Tokoro S, Mori H, Noguchi Y, Morita H, Ivanov II, Sugiyama T, Nuñez G, Camp JG, Hattori M, Umesaki Y, Honda K 2015 Th17 cell induction by adhesion of microbes to intestinal epithelial cells. Cell 163:367–380. doi:10.1016/j.cell.2015.08.058. 26411289 PMC4765954

[33] Kozich JJ, Westcott SL, Baxter NT, Highlander SK, Schloss PD 2013 Development of a dual-index sequencing strategy and curation pipeline for analyzing amplicon sequence data on the MiSeq Illumina sequencing platform. Appl Environ Microbiol 79:5112–5120. doi:10.1128/AEM.01043-13. 23793624 PMC3753973

[34] Myer PR, Kim M, Freetly HC, Smith TP 2016 Evaluation of 16S rRNA amplicon sequencing using two next-generation sequencing technologies for phylogenetic analysis of the rumen bacterial community in steers. J Microbiol Methods 127:132–140. doi:10.1016/j.mimet.2016.06.004. 27282101

[35] Linetzky Waitzberg D, Alves Pereira CC, Logullo L, Manzoni Jacintho T, Almeida D, Teixeira da Silva ML, Matos de Miranda Torrinhas RS 2012 Microbiota benefits after inulin and partially hydrolized guar gum supplementation: a randomized clinical trial in constipated women. Nutr Hosp 27:123–129. doi:10.1590/S0212-16112012000100014. 22566311

[36] Hatamoto M, Kaneko T, Takimoto Y, Ito T, Miyazato N, Maki S, Yamaguchi T, Aoi T 2017 Microbial community structure and enumeration of *Bacillus* species in activated sludge. J Water Environ Technol 15:233–240. doi:10.2965/jwet.17-037.

[37] Guillen IA, Camacho H, Tuero AD, Bacardí D, Palenzuela DO, Aguilera A, Silva JA, Estrada R, Gell O, Suárez J, Ancizar J, Brown E, Colarte AB, Castro J, Novoa LI 2016 PCR conditions for 16S primers for analysis of microbes in the colon of rats. J Biomol Tech 3:105–112. doi:10.7171/jbt.16-2703-002. PMC4920503 27382362

